# A Data-Driven Approach to State of Health Estimation and Prediction for a Lithium-Ion Battery Pack of Electric Buses Based on Real-World Data

**DOI:** 10.3390/s22155762

**Published:** 2022-08-02

**Authors:** Nan Xu, Yu Xie, Qiao Liu, Fenglai Yue, Di Zhao

**Affiliations:** 1 State Key Laboratory of Automotive Simulation and Control, Jilin University, Changchun 130025, China; nanxu@jlu.edu.cn (N.X.); xieyu19@mails.jlu.edu.cn (Y.X.); liuqiao20@mails.jlu.edu.cn (Q.L.); 2 Vehicle Measurement, Control and Safety Key Laboratory of Sichuan Province, Xihua University, Chengdu 610039, China; 3 Vehicle Energy Efficiency and Carbon Emission Reduction Evaluation Laboratory, National New Energy Vehicle Technology Innovation Center, Beijing 100176, China; yuefenglai@nevc.com.cn

**Keywords:** lithium-ion battery, real-world data, state of health, SOH degradation model

## Abstract

In the era of big data, using big data to realize the online estimation of battery SOH has become possible. Traditional solutions based on theoretical models cannot take into account driving behavior and complicated environmental factors. In this paper, an approximate SOH degradation model based on real operating data and environmental temperature data of electric vehicles (EVs) collected with a big data platform is proposed. Firstly, the health indicators are extracted from the historical operating data, and the equivalent capacity at 25 °C is obtained based on the capacity–temperature empirical formula and the capacity offset. Then, the attenuation rate during each charging and discharging process is calculated by combining the operating data and the environmental temperature. Finally, the long short-term memory (LSTM) neural network is used to learn the degradation trend of the battery and predict the future decline trend. The test results show that the proposed method has better performance.

## 1. Introduction

With the intensification of the global greenhouse effect, all countries are committed to the development of new energy vehicles. Electric vehicles can greatly reduce greenhouse gas emissions, and ownership is increasing rapidly [1]. Coupled with stricter emissions policies and more investment in electric vehicle industries around the world, ownership of electric vehicles is likely to maintain a trend of rapid growth [2]. Lithium-ion power batteries are widely used in electric vehicles due to their high energy density, low self-discharge rate, and long cycle life. The driving range and dynamic performance of an EV are largely dependent on the performance of the battery system [3]. With the continuous improvement of lithium-ion battery manufacturing technology, electric vehicles can have longer driving ranges [4]. Power batteries are equivalent to the “heart” of electric vehicles. Therefore, in order to make better use of electric vehicles, some parameters of power batteries need to be monitored and estimated. Battery management systems (BMSs) are basic devices that control the charging and discharging process of the power battery in an electric vehicle. They can also monitor and estimate some parameters of the battery, such as current, voltage, temperature, state of charge (SOC), and state of health [5]. With the increased usage of power batteries their capacity and power will gradually degrade. The capacity fading indicates that the energy that the battery can store decreases with age, and the power degradation indicates that the internal resistance of the battery increases [6]. The above two phenomena indicate that the power battery has a limited service life and the performance will decrease in the process of use, which is one of the main problems that lithium-ion batteries are still facing [7]. It is very important to accurately estimate the state of health, and this is still an open area in the field of batteries.

There are many methods for estimating SOH. Xiong et al. [8] divided them into two categories: experiment-based methods and model-based methods. Experiment-based methods are simple, but a large number of experiments are needed to obtain the aging parameters. Model-based methods need to obtain parameters such as capacity through identification methods. In order to study the variation law of battery capacity, Patrick Wesskamp et al. [9] conducted a long-term aging study on 120 lithium-ion batteries, analyzed the correlation between battery state, temperature and battery capacity during the entire battery life, and established a dynamic-state space model. However, this model only covers some typical working conditions of vehicle operation, and there may be large errors in the prediction of other working conditions in reality, and the realization of this dynamic-state space model requires an accurate battery model as a basis. Zheng et al. [10] proposed a method for vehicle battery capacity estimation based on incremental capacity analysis (ICA) and differential voltage analysis (DVA). Izaro Laresgoiti et al. [11] explained the aging mechanism of the battery based on the complex parameters calculated by the electrochemical model of the battery. However, this required long-term experiments to obtain the parameters, which is not easy to implement in practical applications.

With the arrival of the information age, big data technology has brought opportunities and challenges to the estimation of battery health. Many cities have begun to use big data and monitoring platforms to collect and analyze real-time operating data of new energy vehicles [12]. Estimating SOH through big data is not only conducive to the supervision of the power batteries being used, but also conducive to the disposal of electric vehicle batteries after scrapping. The big data acquisition system will store massive amounts of data. How to filter out effective data is a key issue in estimating SOH. Moreover, the data collected by big data platforms is different from that collected in laboratory experiments. In the process of real vehicle operation, the working conditions of power batteries will also change greatly due to the changes in driver and driving environments. Many dynamic factors will be increased; however, the performance degradation of the battery is more complicated [13].

Machine learning is an artificial intelligence algorithm that can effectively analyze big data and discover its value [14]. In recent years, a lot of research has focused on using data-driven methods to improve the accuracy of SOH estimation [15]. The data-driven method can avoid the dependence on the battery aging mechanism and the battery model. Phattara Khumprom et al. [16] presented the preliminary development of data-driven prognostics and used deep neural networks (DNNs) and the NASA (PCoE) battery dataset to predict the SoH and the RUL of a lithium-ion battery. Daniel Vieira et al. [17] proposed a general data-driven method for estimating battery SOH. Through continuous experiments on lithium-iron phosphate batteries in the laboratory, the original dataset was obtained, and then a NARX (nonlinear auto-regressive network with exogenous inputs) neural network was explored. However, the data obtained in the experiment were all collected at a temperature of 25 °C, and this method did not take into account the influence of temperature on SOH. The essence of data-driven methods to estimate SOH is using machine learning algorithms to build a map from features to SOH. Wu et al. [18] used the speed and arc length curvature of the battery terminal voltage curve during battery charging as a characteristic variable and combined this with the group method of data handling (GMDH) for the polynomial neural network to estimate the battery SOH. This method can estimate the SOH of different types of lithium-ion batteries. However, this method relies heavily on the accuracy of voltage acquisition and is easily affected by measurement noise during practical applications. There are many characteristics that can indicate battery degradation, such as capacity, internal resistance, surface temperature, etc. The reasonable selection of health characteristics is of great significance to machine learning models. Zhang et.al [19] used LSTM with an attention mechanism (ALSTM) to estimate battery SOH online. The model used the current, voltage and temperature in the battery historical data, and the model was a better fit for the battery degradation trend. However, the historical data used to build the model were high-quality data collected in the laboratory, and were obtained from constant-current charging–discharging experiments at room temperature, which is quite different from battery working conditions in the real environment. Lin et al. [20] proposed a method to estimate SOH using ohmic internal resistance by studying the linear relationship between ohmic internal resistance and battery capacity degradation. Feng et al. [21] used the battery surface temperature as a health feature to estimate the battery SOH. Yang et al. [22] established the relationship between long-term coulombic efficiency and battery degradation rate based on the data collected from the cycle life test. Wei et al. [23] used support vector machines to estimate SOH.

The above methods can accurately estimate SOH, but most of the batteries worked under laboratory conditions and were verified under specific working conditions. In the actual operation of electric vehicles, the working conditions of the battery were much more complicated than those in the laboratory. In order to estimate the SOH of an electric vehicle operating in a real environment, Xiong et al. [24] proposed a battery SOH prediction method based on a moving window using data collected by a real battery management system in an electric vehicle. The error of capacity estimation of this method was less than 1.5%. However, the algorithm did not consider low-temperature conditions and changes in discharge rate enough. Liang et al. [25] proposed a new method for estimating the ohmic resistance based on the circuit model and data-driven algorithm, and then estimated the state of health based on the relationship between the ohmic resistance and the battery SOH. However, the correlation or regression relationship between internal resistance (IR) aging and many other vehicle parameters, such as battery temperature, vehicle speed, etc., has not been explored. She et al. [26] estimated the actual power battery SOH based on the ICA method and verified it on the real vehicle, which had a good estimation effect. However, to obtain effective IC values, full charging and discharging tests and a high sampling frequency were required. This is not easy to obtain for actual vehicles, because there is rarely a complete charging or discharging process in the use of real vehicles. Lewis Driscoll et al. [27] extracted features observed from patterns in the voltage, current and temperature profiles during the charging process. Then, they proposed a simple, yet effective, state of health estimation model and used artificial neural networks for estimation. Compared with the above methods, the method proposed in this paper has the advantages shown in Table 1.

In summary, most of the existing methods for estimating SOH are based on laboratory data. The proposed methods rely on the stability of the laboratory environment and have not been verified with actual vehicles. It is still difficult to accurately estimate the battery SOH of EVs in the real environment. On the one hand, a power battery in an actual vehicle cannot be measured in the same way as a battery in a laboratory. On the other hand, the actual vehicle estimation increases the influence of dynamic factors such as the driver. Therefore, we need to study a more accurate capacity measurement method that can be used in real vehicles, and then analyze how the increased dynamic factors affect the change in SOH.

When using big data for real-time SOH estimation, we need to consider both time and accuracy. In order to reduce the training time of the prediction model, it is necessary to extract features highly related to SOH. Therefore, this paper develops an approximate SOH degradation model in the preprocessing stage, which can provide simple and effective training data for the prediction model, and can predict the changing trends of SOH with a simple neural network configuration.

This paper proposes a SOH estimation method that can be directly used in real vehicles. The main contributions can be summarized as follows: (1) Improving a battery capacity calculation method suitable for low-frequency data that can extract aging characteristics from each charging process of EVs. (2) Proposing a method for establishing a SOH approximate attenuation model considering dynamic factors such as driver and ambient temperature. (3) Proposing a method that can predict the remaining service life of the battery by using the LSTM network and the established model. The model is verified by predicting data for 4 months, and the predicted effect of the model is good.

The rest of this paper is arranged as follows: Section 2 demonstrates the data description and preprocessing process. Section 3 describes the calculation of the aging characteristics and the influence of temperature on aging indicators. Section 4 demonstrates the construction method of the SOH approximate degradation model. Section 5 describes the use of the LSTM neural network to predict remaining useful life (RUL), and shows the predicted results, followed by the conclusions in Section 6.

## 2. Data Description and Preprocessing

**Figure 1:**
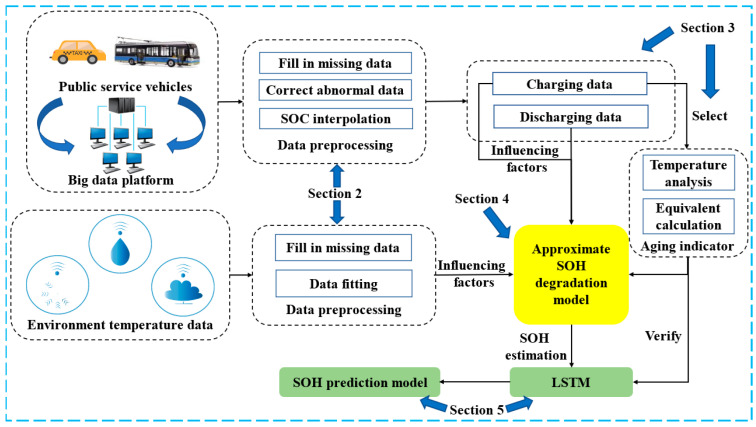
The flow chart of SOH estimation and prediction based on real-world data.

**Figure 2:**
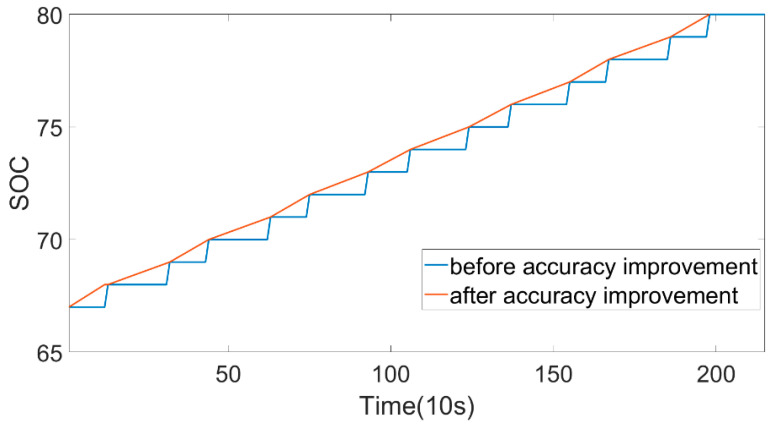
Comparison before and after SOC accuracy improvement.

The flow chart of SOH estimation and prediction based on big data is demonstrated in Figure 1. The vehicle data were derived from the New Energy Vehicle National Supervision Platform, and the operating data were collected from May 2019 to September 2020. The driving range was 4799 km~68,644.9 km. Through vehicle-to-platform communication, the data sampling interval of the monitored car was 10 s. The vehicle studied in this paper is a pure electric bus (North BFC6109GBEV5 model), and the power battery pack was composed of 372 battery cells which had a rated capacity of 404 Ah and a rated pack voltage of 598.92 V. The structure of this power battery was 186 cells in series and 2 cells in parallel. The battery pack had 64 temperature probes and 200 voltage probes to monitor the temperature and voltage of the cells. The accuracy of SOC was 1%, which caused larger calculation errors when calculating battery capacity using SOC. Therefore, the accuracy of SOC values needs to be improved. The formula to improve accuracy is based on the current integration method, as shown in Equation (1). The SOC after accuracy improvement is shown in Figure 2.

(1) $${\mathrm{SOC}}_{\mathrm{AI}}(k)={\mathrm{SOC}}_{l}+\frac{i(k)*dt(k)}{\sum_{j=1}^{n}i(j)*dt(j)}$$

where ${\mathrm{SOC}}_{l}$ is the initial value when the battery SOC = l, n is the number of sampling points with SOC = l, i is the total current, dt is the sampling time interval, and ${\mathrm{SOC}}_{\mathrm{AI}}(k)$ is the SOC value after accuracy improvement.

**Figure 3:**
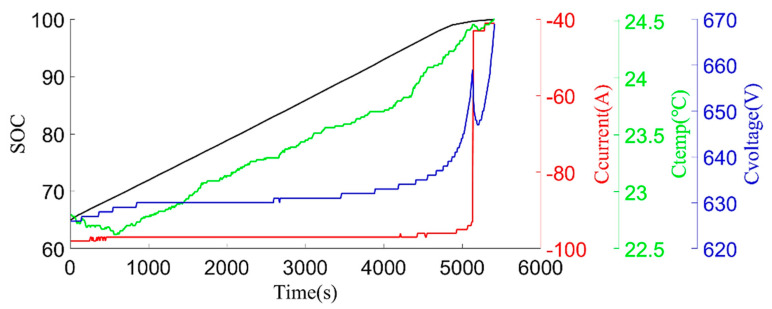
Example of charging process: pack SOC, pack charging current (Ccurrent), pack charging temp (Ctemp), and pack charging voltage (Cvoltage).

**Figure 4:**
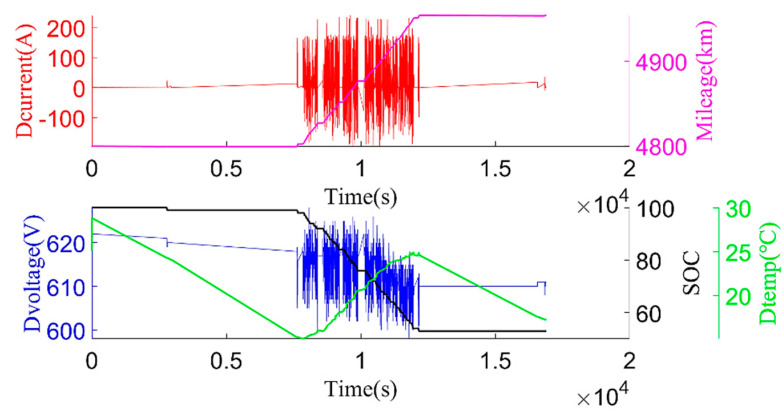
Example of discharging process: pack discharging current (Dcurrent), mileage, pack discharging voltage (Dvoltage), pack SOC, pack discharging temp (Dtemp).

The original data were divided into charging data and discharging data. Some variation parameters of charging phases and discharging phases are shown in Figure 3 and Figure 4.

**Figure 5:**
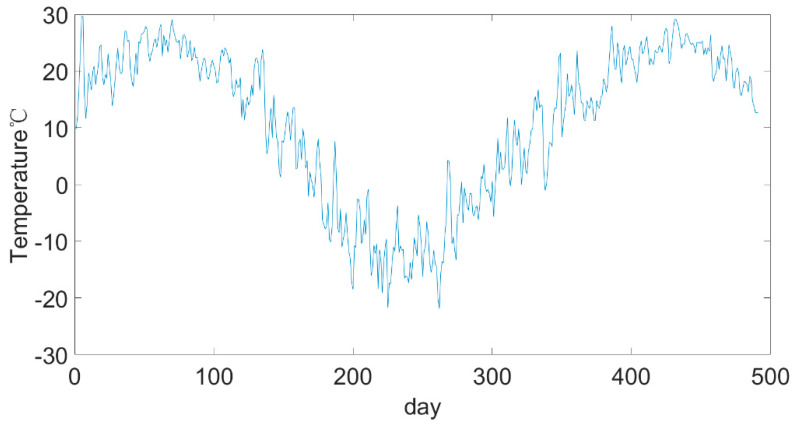
Average daily temperature from May 2019 to September 2020.

The environmental temperature data came from Airwise (hz.zc12369.com, accessed on 11 July 2021), which is a visual display platform of atmospheric environmental data. It can provide hourly weather forecast data for key cities across the country, as well as historical weather data for the past ten years, including temperature, humidity, wind speed, wind level and so on. The bus ran from 5:00 to 23:00. The temperature data during this period of time were extracted from the ambient temperature data, and the average value was calculated to obtain the daily average temperature data from May 2019 to September 2020, as shown in Figure 5. 

## 3. Aging Indicator Calculation and Temperature Influence Analysis

There are many battery aging characteristics. Through the analysis of data, the current capacity of the battery and the average voltage of the battery during charging were selected as the aging characteristics. When calculating the current capacity of the battery, in order to make the calculation more accurate, the charging data were filtered according to the following requirements. 

(1) The SOC had increased by no less than 10%. If the SOC was too small, the overall change would not be reflected, and the calculation error would be larger;

(2) The SOC was not less than 25%, because the unstable battery performance would affect the calculation results if the SOC was too low;

(3) SOC was not higher than 80%; BMS will generally correct SOC when SOC exceeds 80%, resulting in algorithm errors;

(4) The data of the sampling time interval did not exceed 30 s.

Shen et al. [28] proposed that the current capacity can be directly achieved using the difference of SOC within a period of time. Hu et al. [29] applied this method, and the current capacity calculation model is shown in Equation (2).

(2) $$C_{aged}=\frac{\int_{t_{0}}^{t}i(\tau )d\tau }{\Delta \mathrm{SOC}}\approx \frac{\sum_{k=1}^{n}i(k)*dt(k)}{{\mathrm{SOC}}_{t}-{\mathrm{SOC}}_{t_{0}}}$$

where $t_{0}$ is the starting moment, t is the end time, i is totally current, dt is the sampling time, and $C_{aged}$ is the current capacity. In order to avoid errors caused by SOC correction, the upper limit of the charging interval is selected as SOC = 80%. By observing the SOC data when the car is running every day, it was found that more than 90% of the cut-off SOC was concentrated above 65%, so the lower limit of the charging interval was selected as SOC = 65%.

**Figure 6:**
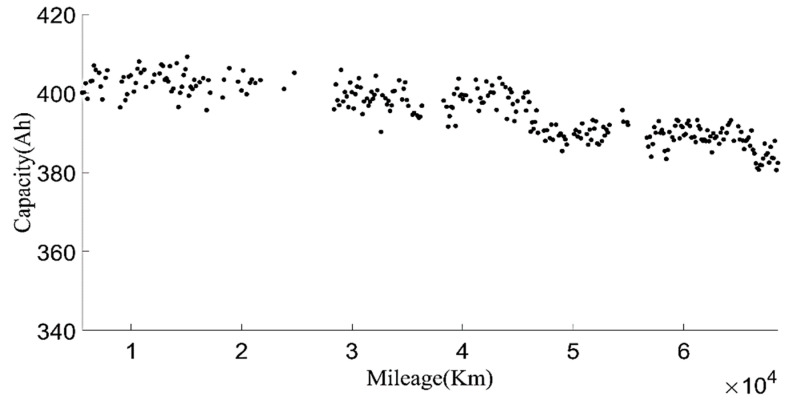
The relationship between the current capacity and the mileage.

In order to make the current capacity calculation more accurate, a variable sliding window algorithm was adopted, and the lengths of the sliding time windows were selected according to the charging current. When the charging current was 100 A, the sliding time window length was 1600 s and the sliding step length was 10 s; when the charging current was 200 A, the sliding time window length was 800 s and the sliding step length was 5 s. For the capacity value calculated in each process, the box diagram method was used to eliminate abnormal values, and then the remaining capacity values were averaged as the current capacity calculated in each charging process. Generally, the lifetime of the battery was indicated by the number of cycles. However, in the realistic driving of vehicles, due to the driver’s requirements, the usable range of the battery SOC of electric vehicles changed, and the cut-off SOC of the data varied widely, between 25% and 70%. Using the number of equivalent full cycles to represent battery aging is inconvenient and has little practical significance. The mileage of an electric vehicle is a suitable representation of the total discharging capacity of the battery, so the accumulated mileage is used to evaluate the aging degree of the battery [30]. Figure 6 shows the relationship between the present battery capacity and mileage.

**Figure 7:**
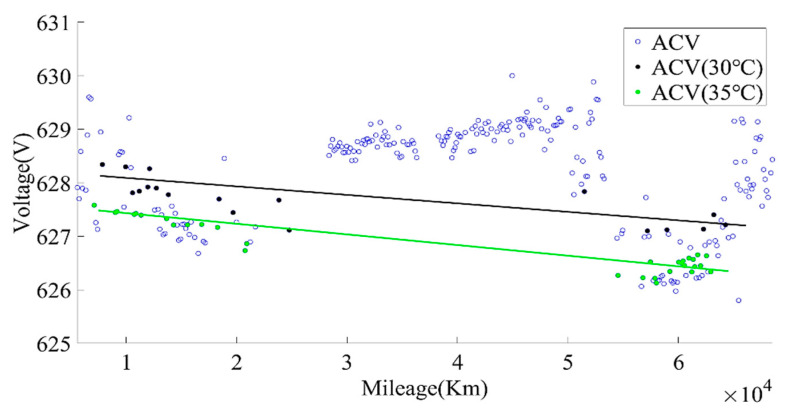
The relationship between pack voltage and the mileage.

The SOC range for calculating the current capacity was selected, as above, and then the average charging voltage within this range was calculated, as shown in Figure 7. It can be found from Figure 7 that the average charging voltage varied greatly due to temperature. By screening the average charging voltage at 30 °C and 35 °C, the data showed a significant downward trend, which can be used as an indicator for evaluating battery aging. It should be noted that the charging current also had a great impact on the change in the average charging voltage of the battery. When calculating the average charging voltage (ACV), the charging current of the same rate should be selected.

**Figure 8:**
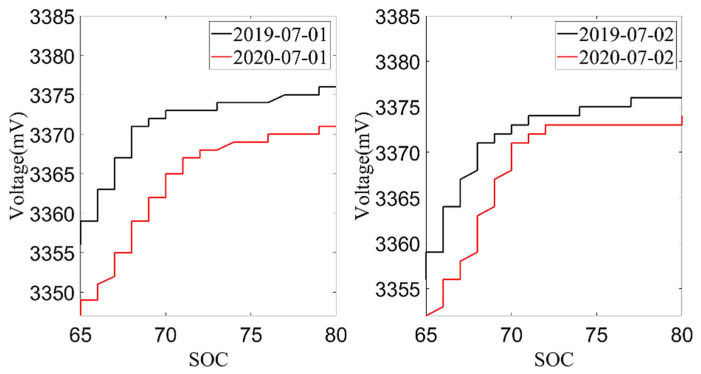
The relationship between SOC and cell voltage.

**Figure 9:**
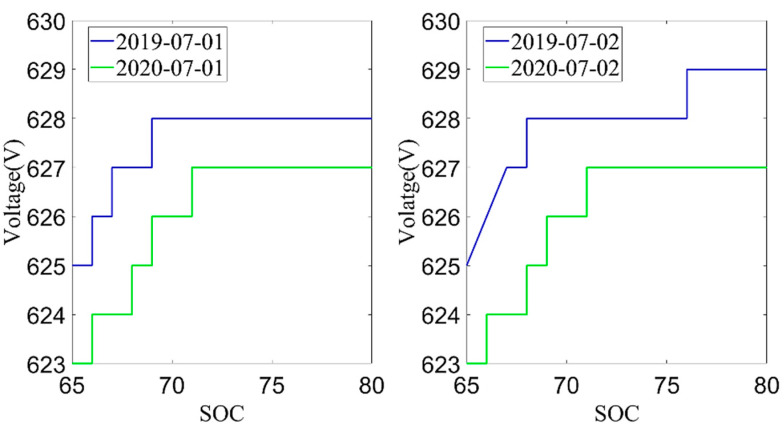
The relationship between SOC and pack voltage.

Next, the average charging voltage in July 2019 and July 2020 was analyzed. The data from July 1st and 2nd were selected according to the environmental temperature, charging current and charging temperature conditions, as shown in Figure 8 and Figure 9. From these two figures, it can be found that the trend of the cell voltage and the total battery voltage was consistent. When the battery SOC reached the same value, the charging voltage in July 2020 was lower than the charging voltage in July 2019. The decrease in battery charging voltage also reflects the increase in battery internal resistance, which is also an important indicator for evaluating the battery aging. However, the average charging voltage was much more affected by temperature than the current capacity, so the current capacity of the battery is used as the index to evaluate the battery SOH in this paper.

**Figure 10:**
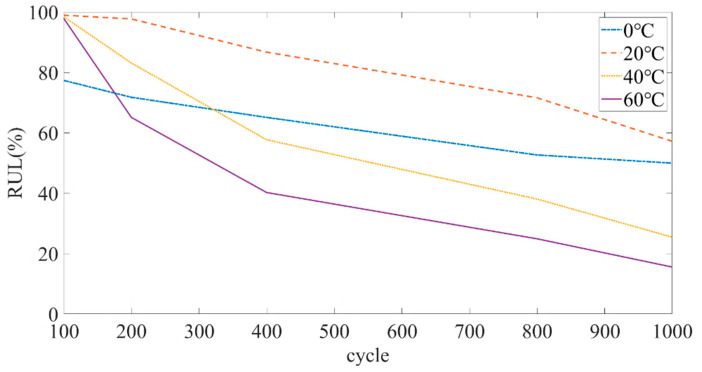
Degradation rate at different temperatures.

Hwabhin Kwon et al. [31] have studied the relationship between the current capacity of lithium iron phosphate batteries and the number of cycles at different temperatures, and found that temperature is a key factor affecting the performance and durability of lithium-ion batteries. Figure 10 shows the degradation rate of the lithium iron phosphate battery at different temperatures calculated based on the extracted data. It can be found from Figure 10 that: (1) The degradation rate of the battery was different at different temperatures. (2) When the battery was cycled a certain number of times at the same temperature, the degradation rate also changed. (3) The SOH degradation rate of the battery was lower when it was around 20 °C. In addition, the battery attenuated faster at a high temperature (40 °C) than at a low temperature (0 °C).

**Figure 11:**
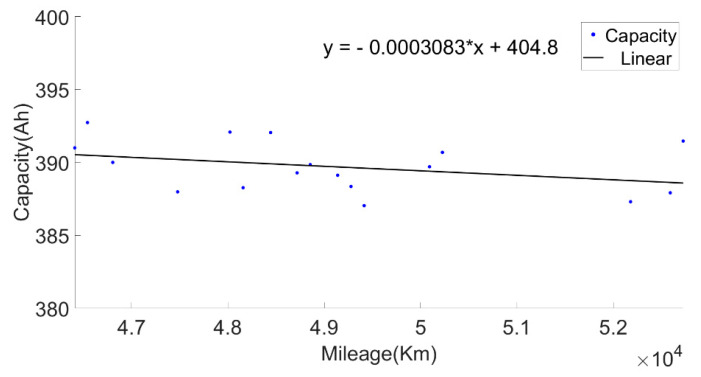
The relationship between the current capacity and the mileage at charging temperature of 23 °C.

**Figure 12:**
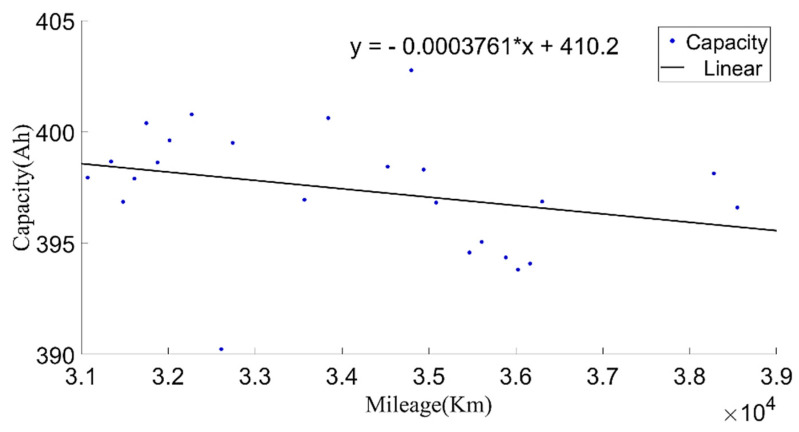
The relationship between the current capacity and the mileage at the charging temperature of 25 °C.

**Figure 13:**
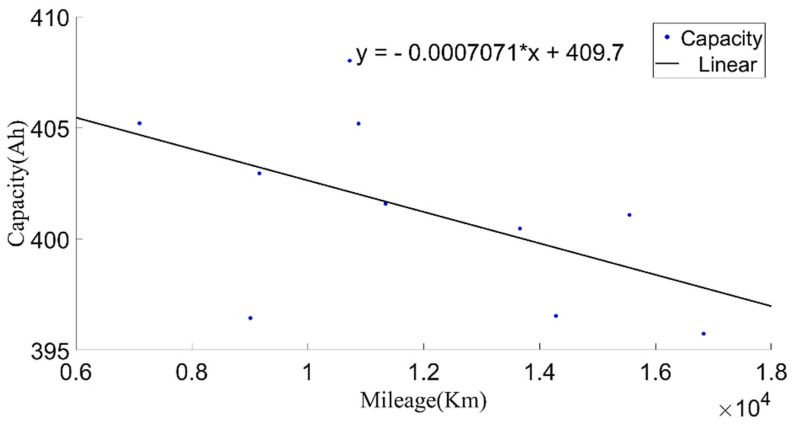
The relationship between the current capacity and the mileage at the charging temperature of 35 °C.

**Figure 14:**
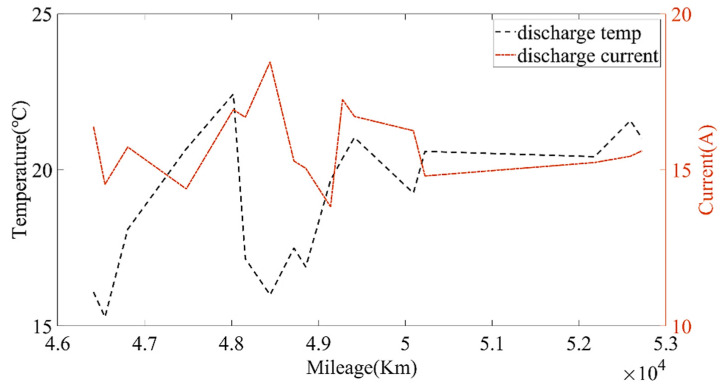
The relationship between the discharging temperature, discharging current and the mileage at the charging temperature of 23 °C.

**Figure 15:**
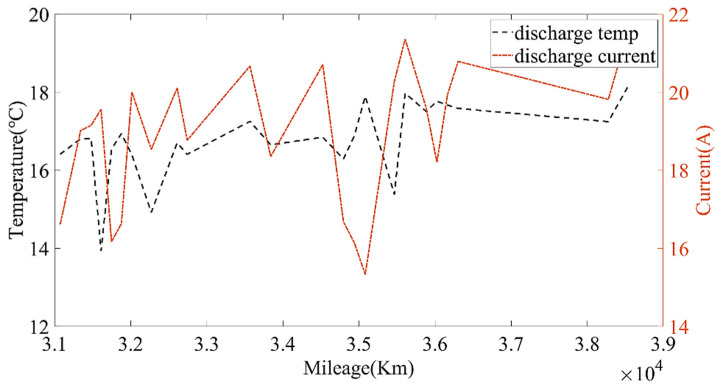
The relationship between the discharging temperature, discharging current and the mileage at the charging temperature of 25 °C.

**Figure 16:**
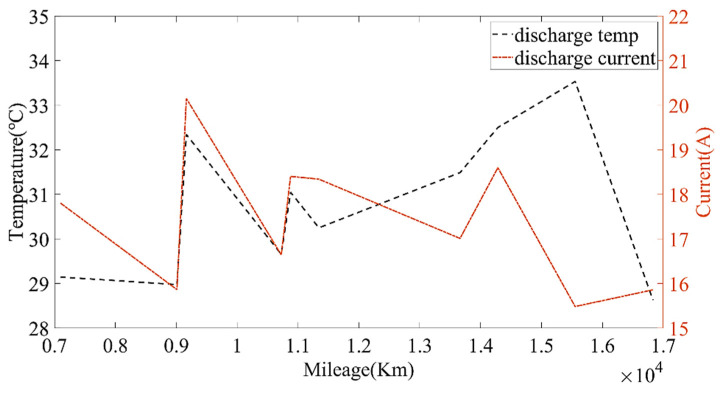
The relationship between the discharging temperature, discharging current and the mileage at the charging temperature of 35 °C.

Then, the calculated current capacity data were filtered based on the average charging temperature. Figure 11, Figure 12 and Figure 13 show the capacity at the average charging temperatures of 23 °C, 25 °C and 35 °C. In the spring, the average charging temperature of the battery was 23 °C, falling into the normal temperature operation section for electric buses. It can be seen from Figure 14 that the average discharging temperature of the battery was between 18 and 23 °C. The data showing the battery charging at 25 °C were mainly acquired in winter (the battery will be heated before charging in winter, so the average charging temperature of the battery in winter will be higher than that in the spring). This can be used as a low-temperature operation section, as shown in Figure 15. The average discharging temperature of the battery was between 14 and 18 °C. The data that show the battery charging at 35 °C were mainly acquired in the summer, which is a high-temperature operation section. As shown in Figure 16, the average discharging temperature of the battery was between 29 and 32 °C. According to the previous analysis, the battery had the lowest degradation rate at about 20 °C, and had a lower degradation rate at low temperature than at high temperature. It can be seen from Figure 12 and Figure 13 that the battery degradation rate was consistent with the previous analysis. 

Figure 14, Figure 15 and Figure 16 show that the average discharging current of the battery in different seasons was different. The average discharging current in spring was lower than the average discharging current in summer and winter, which may be caused by the frequent use of air conditioning in hot and cold weather. The change in the discharging current can reflect the operating habits of the driver to a certain extent, and the discharging rate also has a great impact on the SOH. Therefore, when considering the factors that affect the state of health, the discharging current needs to be taken into account as a parameter.

By analyzing the relationship between capacity attenuation and mileage at different temperatures, it was found that the initial capacity varied at different temperatures. This reflects the influence of temperature on the change in battery capacity. Based on the acquired data, the relationship between the capacity change and temperature was analyzed. We found that the empirical Formula (3) for temperature and battery capacity change could not fully indicate the capacity change characteristics of the studied vehicle with temperature. Therefore, an equivalent capacity calculation method based on the combination of empirical formula and capacity offset is proposed as follows:

(3) $$Q_{t2}=\frac{Q_{t1}}{1+K(t_{1}-t_{2})}$$

where *K* is the temperature coefficient; $t_{1}$ and $t_{2}$ are battery temperature; and $Q_{t1}$ and $Q_{t2}$ are the corresponding battery capacities.

**Figure 17:**
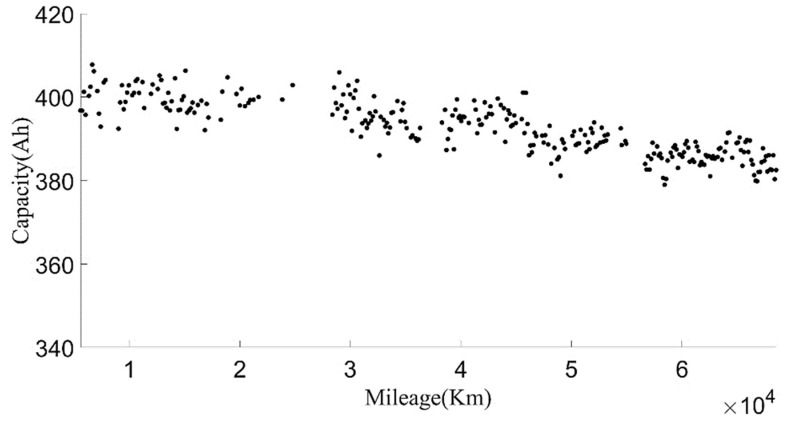
The relationship between the equivalent current capacity (at 25 °C) and the mileage.

Through the analysis of the calculated battery capacity and environment temperature, it can be found that the calculated battery capacity in cold weather was higher than that in warm weather; this may be caused by battery heating mechanism. This phenomenon is defined as capacity offset ΔC. At other temperatures, the empirical formula can be used to calculate the equivalent capacity. The temperature coefficient K was calculated from the initial capacity at 35 °C and 23 °C. Then, we used Equation (4) to calculate the equivalent capacity $C_{e}$ at 25 °C. The variation in equivalent capacity with mileage is shown in Figure 17.

(4) $$C_{e}=\left\{ \begin{array}{l} C_{aged}-\Delta C,Dtemp\leq 18℃\\ \frac{C_{aged}}{1+K(t_{1}-25)},Dtemp>18℃ \end{array}\right.$$

where ΔC is the capacity offset in this paper; ΔC = (410.2 − 405.9534) Ah = 4.2466 Ah; and Dtemp is the average discharging temperature.

## 4. Approximate SOH Degradation Model

SOH is used to describe the health of a battery. There are many ways to define SOH, such as battery capacity, the internal resistance of the battery, and cycle times. According to the literature [32,33], SOH is defined by the ratio of current capacity to initial capacity in this paper, as shown in Equation (5).

(5) $$\mathrm{SOH}=\frac{C_{aged}}{C_{0}}\times 100\%$$

where $C_{0}$ is the initial capacity and $C_{aged}$ is the current capacity.

When ignoring the influence of environmental temperature on SOH, the relationship between battery SOH degradation and operating conditions can be expressed as “battery SOH degradation = battery operating state × battery operating time” [34]. The battery operating state is divided into the battery charging state and the battery discharging state. The charging current and the discharging current are used as the characteristic values of the battery operating state during the driving and charging of the car, respectively, and the charging time and the discharging time are regarded as the battery operating time. On this basis, the influence of temperature on SOH is considered. Environmental temperature data are used in the discharging process. In the charging process, the charging temperature data are used because the battery has heating measures during low-temperature charging. Based on the above analysis, an approximate SOH degradation model under the real operating environment of the battery is obtained, as shown in Equation (6).

(6) $$\begin{array}{ll} \Delta \mathrm{SOH}(i)= & \left((current_{c}(i)*\delta_{1}+temp_{c}(i)*\delta_{2})*time_{c}(i\right)\\ & +(current_{d}(i)*\delta_{3}+temp_{d}(i)*\delta_{4})*time_{d}(i))\\ & /(\sum \left(current_{c}(i)*\delta_{1}+temp_{c}(i)*\delta_{2})*time_{c}(i\right)\\ & +\sum \left(current_{d}(i)*\delta_{3}+temp_{d}(i)*\delta_{4})*time_{d}(i\right))\\ & \;*\Delta \mathrm{SOH} \end{array}$$

where $current_{c}$ is the normalized average charging current, $current_{d}$ is the normalized average discharging current, $temp_{c}$ is the normalized battery temperature during charging, $temp_{d}$ is the normalized environmental temperature, $time_{c}$ is charging time, $time_{d}$ is discharging time, $\delta_{i}$ indicates the weight of different factors, and ΔSOH is the degree of battery degradation over a period of time.

### 4.1. Weight Analysis

**Figure 18:**
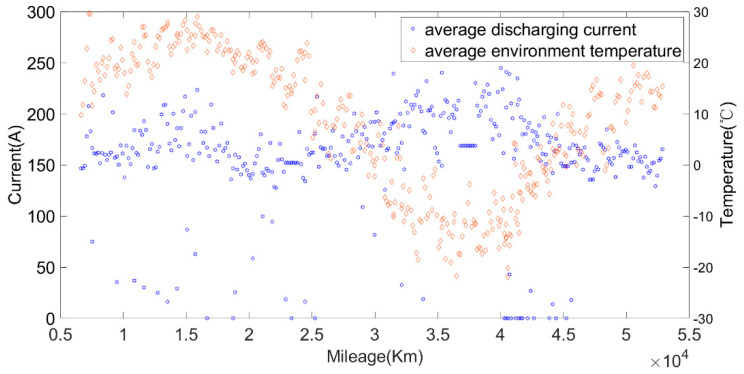
The relationship between the discharging current, average environmental temperature and mileage.

**Figure 19:**
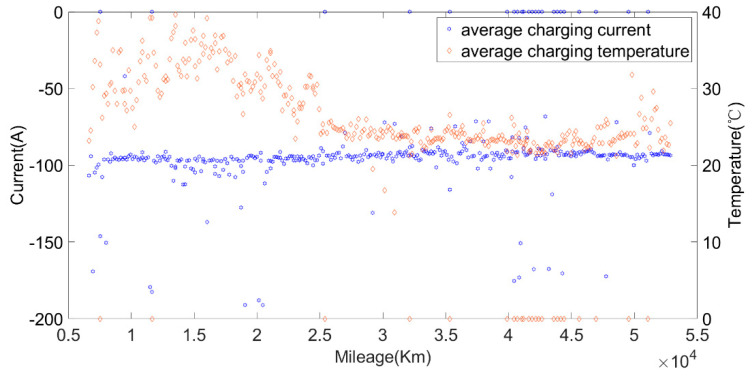
The relationship between the charging current, average charging temperature and mileage.

Li Zhe of Tsinghua University once established the durability model of lithium iron phosphate batteries by studying the effects of different factors on battery SOH, and put forward the concept of “equivalent stress increment” [35]. The weight is determined according to the varied range of different influencing factors $\delta_{i}$. The changes in each factor are shown in Figure 18. It can be seen from Figure 18 that the average discharging current of the car was larger when the environmental temperature was high (in summer) and when the environmental temperature was low (in winter). In addition, according to the discharging data mentioned above, it can be seen that the peak discharging current of the battery was almost within 250 A. It can be seen from Figure 19 and the charging data provided in the previous article that most battery charging was constant current charging, the charging current was stable at around 100 A, and a small part of the charging current was 200 A. Through the above analysis, we found that the battery charging and discharging current would not exceed 1C, so $\delta_{1}$ and $\delta_{3}$ were set to 1. According to the results of the literature [35], the degradation rate doubled for every 10 °C increase in temperature, so we set $\delta_{2}$ and $\delta_{4}$ as Equation (7).

(7) $$\left\{ \begin{array}{l} \delta_{2}=1+\frac{\left|temp_{c}-20\right|}{10}\\ \delta_{4}=1+\frac{\left|temp_{d}-20\right|}{10} \end{array}\right.$$

### 4.2. ΔSOH Calculation

In order to avoid the impact of environmental temperature and charging current on capacity calculation, data with similar environmental temperatures were selected from the data with an average charging temperature of 25 °C and a charging current of 100 A. By comparing the temperature data in the charging time period, the data from 20 May 2019 were selected as the starting value of model calculation, and the data from 24 May 2020 were selected as the ending value of model calculation. Using the method mentioned above, the battery capacity at the beginning (${\mathrm{SOH}}_{\mathrm{start}}$) was 403.9106 Ah, the starting mileage was 6482 Km, the battery capacity at the ending time (${\mathrm{SOH}}_{\mathrm{terminal}}$) was 389.2958 Ah, and the ending mileage was 52,862.1 Km. According to the approximate linear degradation law of the lithium iron phosphate battery, the actual factory capacity of the battery (${\mathrm{SOH}}_{\mathrm{initial}}$) was calculated to be 405.9534 Ah, and the calculated capacity was very close to the rated capacity. The result shows that the proposed algorithm for calculating the current capacity had better accuracy. ΔSOH was calculated by Equation (8), ΔSOH=0.03601.

(8) $$\Delta \mathrm{SOH}=\frac{{\mathrm{SOH}}_{\mathrm{start}}{-\mathrm{SOH}}_{\mathrm{terminal}}}{{\mathrm{SOH}}_{\mathrm{initial}}}$$

**Figure 20:**
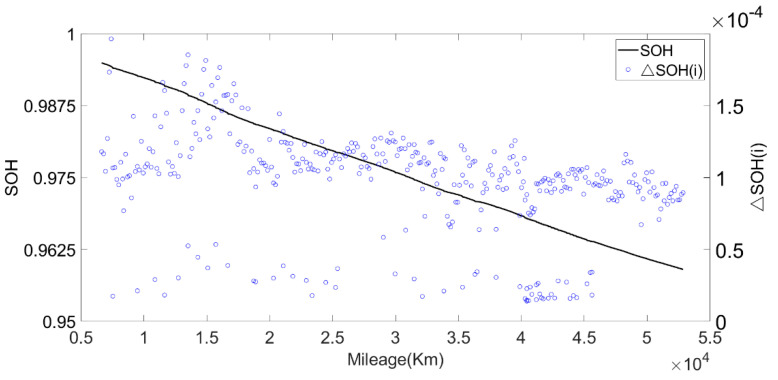
The relationship between SOH, ΔSOH and mileage.

**Figure 21:**
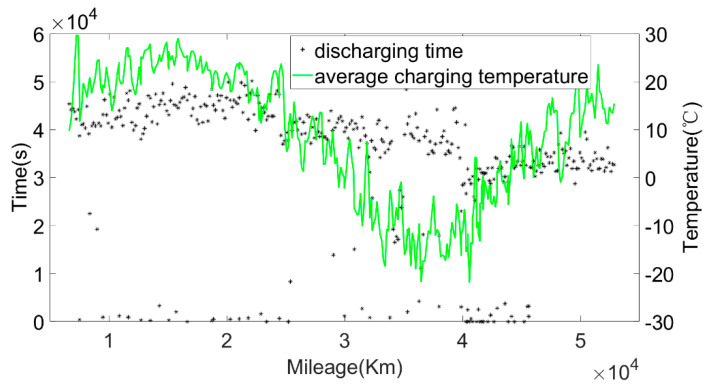
The relationship between discharging time, average charging temperature and mileage.

Next, we calculated ΔSOH(i) and the result is shown in Figure 20. It can be seen from Figure 20 and Figure 21 that the battery degraded faster at high temperature, followed by low temperature and normal temperature.

**Figure 22:**
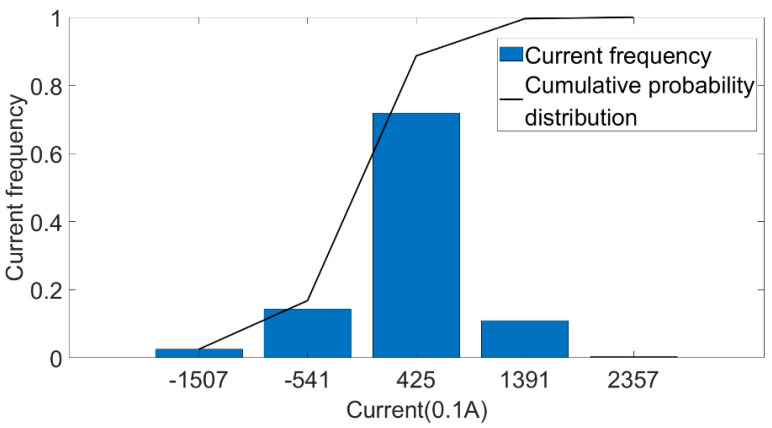
Current frequency distribution.

We conducted a statistical analysis on the measured discharging current data of the No. 361 pure electric bus in Changchun during one year of operation. The data included the discharging current data of the line in different operation periods (morning peak, evening peak and normal period). The discharging current was divided into five sections according to the occurrence frequency, and the results are shown in Figure 22. The frequencies were 2.5%, 14.3%, 71.9%, 10.9%, 0.4%.

**Figure 23:**
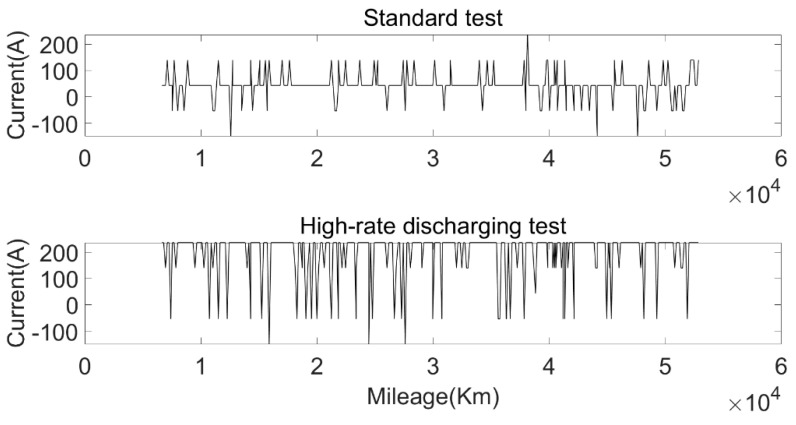
Variation in current with mileage under standard test environment and high-rate current test environment.

**Figure 24:**
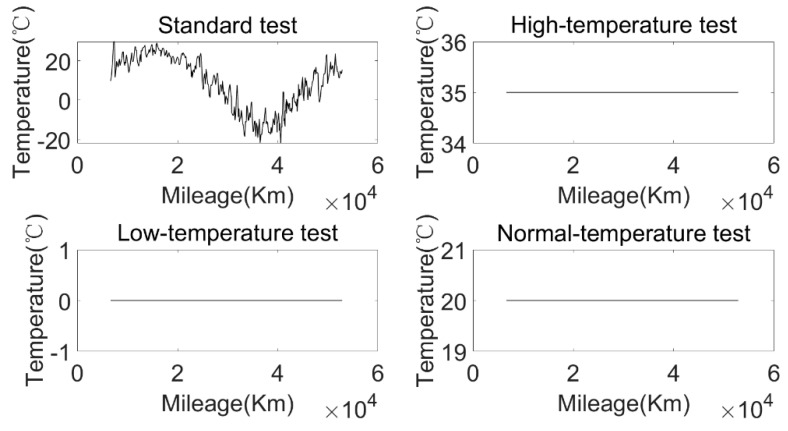
The relationship between environmental temperature and mileage under four different test environments.

**Figure 25:**
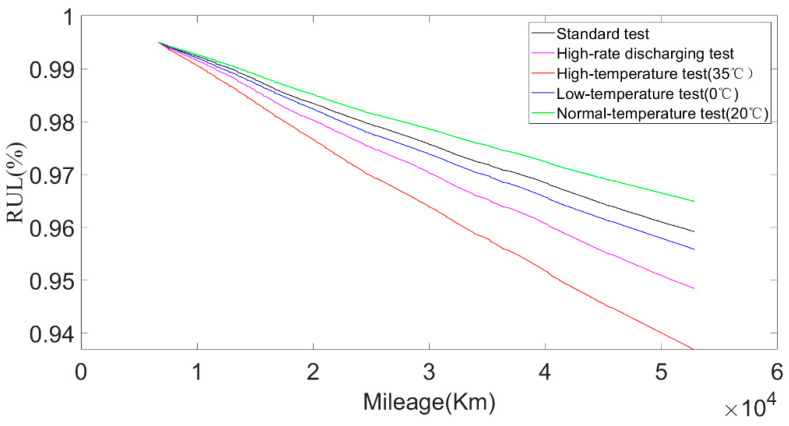
RUL calculation results of battery under different test environments.

According to the environmental temperature and discharging current, the battery test conditions were divided into five different test environments: a standard test environment, a high-rate discharging test environment, a high-temperature test environment, a normal-temperature test environment, and a low-temperature test environment. The discharging current and environmental temperature under different test environments are shown in Figure 23 and Figure 24. The standard current refers to the discharging current distributed according to the statistical proportion, and the standard temperature was the average environmental temperature during the operation of the bus over one year. The calculated SOH degradation rate of the approximate SOH degradation model under five test environments is shown in Figure 25.

It can be seen from Figure 25 that the battery had a lower degradation rate in the normal-temperature test environment, and had a higher degradation rate in the high-temperature test environment and the high-rate current test environment. In addition, the degradation rate in the high-temperature test environment was higher than that in the low-temperature test environment, which is consistent with the previous analysis of the battery degradation law.

## 5. RUL Prediction Model Based on LSTM

**Figure 26:**
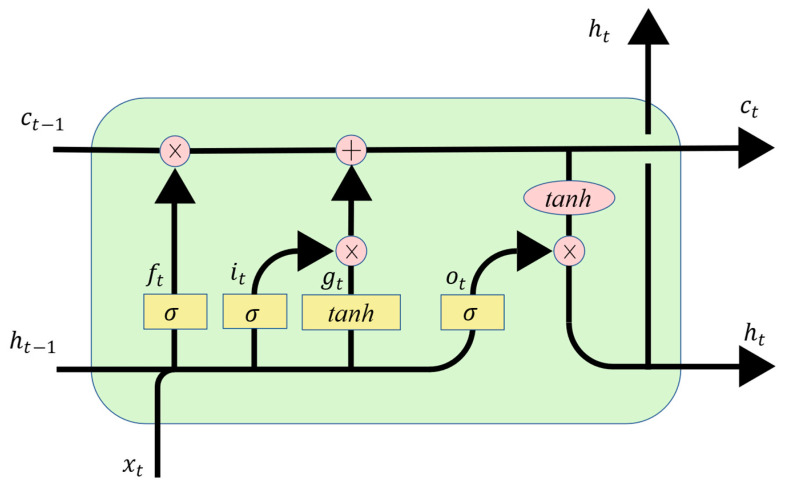
The cell structure of LSTM.

Due to the advantages of LSTM in time series prediction, LSTM has been widely used in battery remaining useful life prediction [36,37]. The LSTM neural network is a variant of the RNN that can solve the problem of gradient disappearance or gradient explosion in RNNs. The basic structure of LSTM is shown in Figure 26; it mainly includes three units: input gate, forget gate and output gate. 

The first unit of LSTM is the forget gate $f_{t}$, and it is able to discard redundant information, which is processed by the sigmoid function to a value of 0 to 1. The gates are calculated as follows:

(9) $$f_{t}=\sigma (W_{x}^{f}x_{t}+W_{h}^{f}h_{t-1}+b^{f})$$

The second gate is the input gate $i_{t}$; it is able to select key information to be stored in the internal state. The key information includes two parts, one is:

(10) $$i_{t}=\sigma (W_{x}^{i}x_{t}+W_{h}^{i}h_{t-1}+b^{i})$$

This part can be seen as how much information of the current input needs to be saved to the unit state. The other part is:

(11) $$g_{t}=tanh(W_{x}^{g}x_{t}+W_{h}^{g}h_{t-1}+b^{g})$$

This part is used to add new information generated by the current input to the unit state. These two parts produce a new memory state. Through the forget gate and input gate, the cell state can be calculated:

(12) $$c_{t}=f_{t}*c_{t-1}+i_{t}*g_{t}$$

The last unit is the output gate $o_{t}$; it is used to calculate the degree to which the information is output at the current moment. Similar to the previous steps, the output gate is calculated as follows:

(13) $$o_{t}=\sigma (W_{x}^{o}x_{t}+W_{h}^{o}h_{t-1}+b^{o})$$

Finally, the hidden state $h_{t}$ is computed by

(14) $$h_{t}=o_{t}*tanh(c_{t})$$

**Figure 27:**
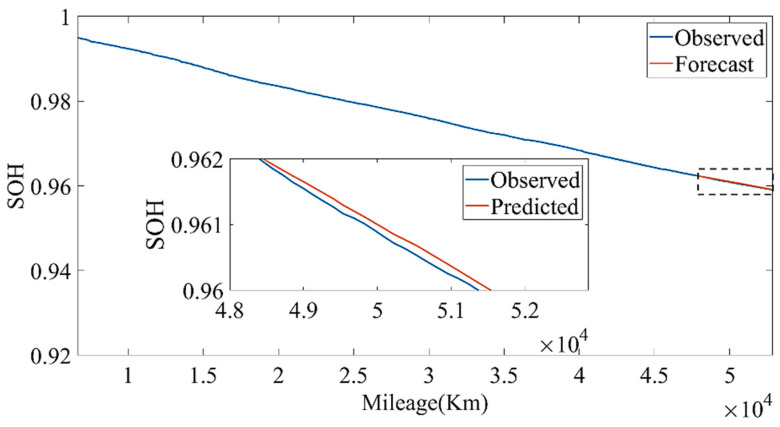
The SOH estimation result based on LSTM.

**Figure 28:**
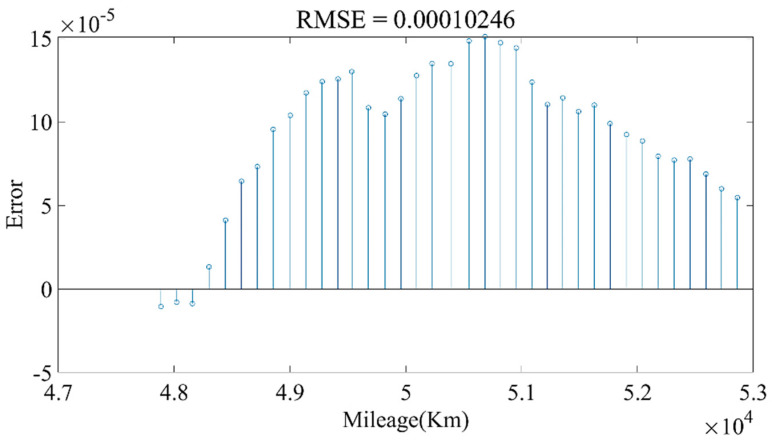
The RMSE of LSTM.

**Figure 29:**
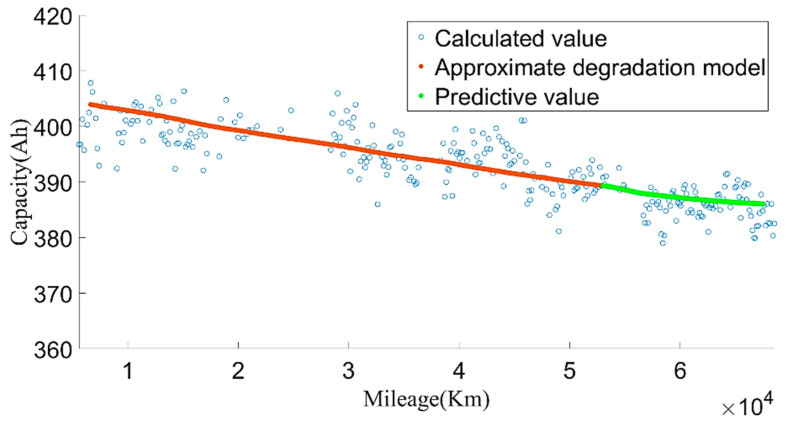
The SOH prediction results based on LSTM.

**Figure 30:**
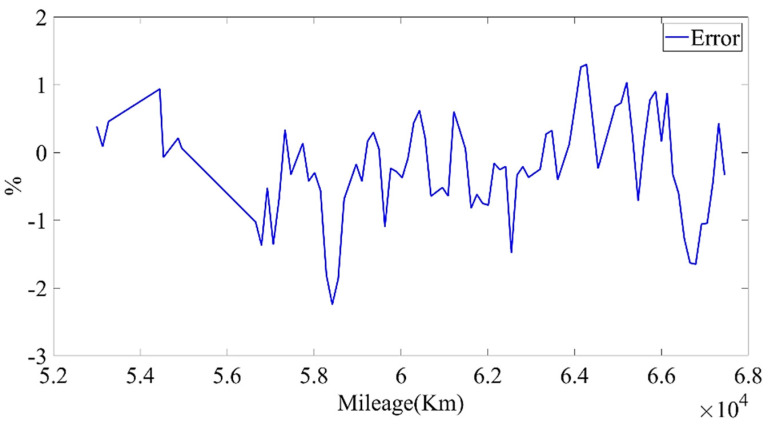
The prediction errors of the LSTM model.

We used the data from 20 May 2019 to 24 May 2020 to establish the model and divided the training set and test set according to the ratio of 9:1. Through constant parameter adjustment of the model, it was found that the neural network had two-layer LSTM with better fitting and prediction effects. The number of hidden neurons in the first layer was 160, and the number of hidden neurons in the second layer was 138. Figure 27 and Figure 28 show the estimated result of the model under this parameter; the root mean square error (RMSE) was 0.010246%. Then, we used the model to predict the data for the next four months. The model prediction results and prediction errors are shown in Figure 29 and Figure 30, respectively. It can be seen from Figure 30 that the absolute value of the model prediction error did not exceed 3%, and most were within 1%.

**Figure 31:**
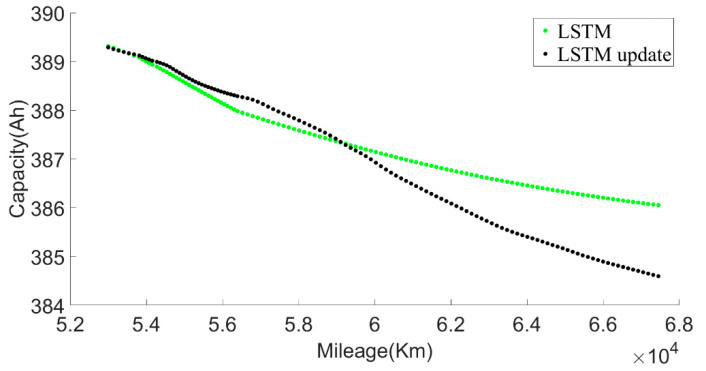
Comparison of prediction results before and after LSTM update.

Figure 29 shows that, as the forecast time increases, the model forecast trend tends to be flat, which will lead to greater and greater forecast errors. Therefore, we set out to update the LSTM model with observations. The observed values were obtained by the approximate SOH degradation model, and the network updated with the observed values was used for prediction. By comparing the prediction un-updated results, it could be found that the updated network prediction trend was more in line with the SOH degradation law, as shown in Figure 31.

The LSTM update represented a retrained model after adding new data. Since the existing battery life was not long enough to cover the entire life cycle, there will be large errors in the long-term prediction of battery RUL. LSTM adopts an offline update method, and can choose to run updates at regular intervals, such as once a month, using the proposed approximate SOH degeneration model to calculate the newly acquired monthly data. The calculated SOH values were input into the LSTM model as new training data, and the battery aging model was gradually completed throughout the life cycle.

## 6. Conclusions and Outlook

This paper has proposed an improved battery capacity calculation method. An effective health indicator (the current capacity) based on partial charging data curves was extracted. Since the change in the calculated capacity value was obviously affected by the temperature, an equivalent capacity based on the temperature capacity empirical formula and capacity offset was calculated. The capacity value after equivalence showed an obvious degradation law. Furthermore, an SOH approximate degradation model was established by analyzing factors such as environmental temperature and charging and discharging current, and the weights of different influencing factors were analyzed in detail. The approximate degradation model could obtain an accurate degradation estimation for battery state of health in each charging and discharging process. Finally, a RUL prediction model based on LSTM was established. The prediction results of the data for the next 4 months showed that the proposed model could achieve high accuracy. 

We will continue to study how to further improve the accuracy of the approximate SOH degradation model, considering more relevant factors such as SOC, vehicle speed, etc. In the next stage, we will develop a generic model that can estimate the battery SOH of different vehicles online based on a big data platform while predicting the trends of SOH changes at the same time. 

## Figures and Tables

**Table 1:** The advantages of the proposed method.

Index	Advantage
[24]	Low-temperature ambient conditions are considered.
[25]	No need to consider complex circuit models.
[26]	Feature extraction with only partial charge curves and lower data sampling frequency.
[27]	Features are extracted from real-world data with relatively poor quality, and verified with real-world data

## Data Availability

Not applicable.
